# Investigating Brain Network Changes and Their Association With Cognitive Recovery After Traumatic Brain Injury: A Longitudinal Analysis

**DOI:** 10.3389/fneur.2020.00369

**Published:** 2020-06-09

**Authors:** Nádia Moreira da Silva, Christopher J. A. Cowie, Andrew M. Blamire, Rob Forsyth, Peter Neal Taylor

**Affiliations:** ^1^CNNP Lab, Interdisciplinary Complex Systems Group, School of Computing, Newcastle University, Newcastle upon Tyne, United Kingdom; ^2^Faculty of Medical Sciences, Institute of Neuroscience, Newcastle University, Newcastle upon Tyne, United Kingdom; ^3^Department of Neurosurgery, Newcastle upon Tyne Hospitals NHS Foundation Trust, Newcastle upon Tyne, United Kingdom; ^4^Institute of Cellular Medicine, Newcastle MR Centre, Newcastle University, Newcastle upon Tyne, United Kingdom

**Keywords:** TBI, longitudinal, graph theoretical analysis, network, structural connectivity

## Abstract

Traumatic brain injury (TBI) can result in acute cognitive deficits and diffuse axonal injury reflected in white matter brain network alterations, which may, or may not, later recover. Our objective is to first characterize the ways in which brain networks change after TBI and, second, investigate if those changes are associated with recovery of cognitive deficits. We aim to make initial progress in discerning the relationships between brain network changes, and their (dys)functional correlates. We analyze longitudinally acquired MRI from 23 TBI patients (two time points: 6 days, 12 months post-injury) and cross-sectional data from 28 controls to construct white matter brain networks. Cognitive assessment was also performed. Graph theory and regression analysis were applied to identify changed brain network metrics after injury that are associated with subsequent improvements in cognitive function. Sixteen brain network metrics were found to be discriminative of different post-injury phases. Eleven of those explain 90% (adjusted *R*^2^) of the variability observed in cognitive recovery following TBI. Brain network metrics that had a high contribution to the explained variance were found in frontal and temporal cortex, additional to the anterior cingulate cortex. Our preliminary study suggests that network reorganization may be related to recovery of impaired cognitive function in the first year after a TBI.

## Introduction

Traumatic brain injury (TBI), which frequently involves white matter connectivity damage, is the leading cause of morbidity, death among children, and individuals under the age of 45 (1, 2). Every year in England and Wales, around 1.4 million patients attend hospital after sustaining a recent head injury, which represents 10% of all emergency admissions (3). Although, mild TBI (mTBI) patients usually make good recoveries, a significant proportion experience persistent cognitive deficits (4).

Diffuse axonal injury (DAI), one of the most common pathologies in TBI (5, 6), is triggered by mechanical disruption of axons, resulting in complex, and diverse effects on brain function

(7). Diffusion tensor imaging (DTI) is particularly suited to the study of DAI and has been used to investigate white matter brain connectivity changes after TBI (8, 9). To understand how widespread DAI lesions affect brain function, it may be necessary to analyze the global impact of these lesions on the whole-brain network (9).

Interpreting widespread network changes is challenging. Network neuroscience techniques (e.g., graph theory) allow the description and analysis of human brain network properties and have successfully been applied in cognitive neuroscience (9–11). Those techniques used anatomically defined gray matter regions as nodes and a measure of association (e.g., number of tracts, mean diffusion along tracts, etc.) between pairs of nodes based on the white matter tracts or edges connecting them (12). Those tracts can be estimated from tractography techniques or extracted from white matter atlases. All pairwise associations between regions are compiled in a connectivity matrix. The approach offers alternative analysis of how brain network changes may lead to cognitive variability over time.

Despite the importance of understanding temporal variability, most TBI studies are cross-sectional in design, which has limited value in understanding longitudinal recovery (13). Since TBI is a heterogeneous disorder with a dynamic behavior, longitudinal studies are vital to capture brain changes over time, and establish *longitudinal relationships* (13, 14).

In this study, we identified brain network properties that change over the first 12 months after a TBI using longitudinally acquired data and investigated if these properties relate to changes in cognitive functioning.

## Materials and Methods

### Data Acquisition and Subjects

Twenty-three TBI patients were scanned twice on a 3T MRI scanner (Phillips Achieva MRI) initially a mean of 6 days after injury (early phase) and again 1 year later (late phase). The study protocol consisted of structural T1-weighted sequences [magnetization-prepared rapid-acquisition gradient echo, repetition time (TR) = 8.1 ms, echo time (TE) = 4.6 ms, matrix size 240 × 216 × 180, isotropic 1 mm resolution] and diffusion-weighted images (TR/TE = 2,524/71 ms; 24 slices; *b* = 0; 1000 s mm–^2^; 16 diffusion directions; 2 × 2 × 6 mm^3^ resolution), and it was consistent across all sessions. Acquisition methods have been described previously, as has the effect of injury on the basic DTI-derived metrics in these same participants (15). Patients were classified as mild or moderate TBI based on Glasgow Coma Scale (GCS). Five patients had moderate TBI [Glasgow Comma Sale (GCS): median = 12, IQR = 10–12] and 18 had a mild TBI (GCS: median = 14, IQR = 14–15) (Table 1). Twenty-eight healthy controls matched for age, gender, level of education, and National Adult Reading Test (NART, a proxy for pre-injury educational status) were scanned once. Detailed description of imaging protocols and subject's clinical data (e.g., age, gender, level of education, NART, injury mechanism, Glasgow coma scale, loss of consciousness, and post-traumatic amnesia) can be found in Croall et al. (15) and Extended Data (Tables S1, S2, S3).

**Table 1:** Clinical data of controls and patients with mild or moderate TBI.

**Clinical data**	**Controls**	**Mild TBI patients**	**Moderate TBI patients**	**All TBI patients**	**Controls vs. patients**
GCS	-	Med = 14	Med = 12	Med = 14	-
		IQR = 14–15	IQR = 10–12	IQR = 13.5–15	
LOC (min)	-	Med = 1	Med = 3	Med = 1	-
		IQR = 0–4.5	IQR = 1–10	IQR = 0–5	
PTA (h)	-	Med = 0	Med = 4	Med = 0.5	-
		IQR = 0–1.75	IQR = 1–240	IQR = 0–3	
NART	Med = 112.5	Med = 105.5	Med = 105	Med = 105	*p* = 0.053
	IRQ = 106.8–115	IQR = 92.5–116	IQR = 105–105	IQR = 93–105	
Age	Med = 37.5	Med = 35.5	Med = 38	Med = 36	*p* = 0.933
	IRQ = 27.25–51	IQR = 26.8–48.5	IQR = 24–53	IQR = 25–51	
Level of education	Med = 10	Med = 9	Med = 9	Med = 9	*p* = 0.868
	IRQ = 9–14	IQR = 9–14	IQR = 9–14	IQR = 9–14	
Sex	22 (M)	15 (M)	3 (M)	18 (M)	*p* = 1
	6 (F)	3 (F)	2 (F)	5 (F)	

*The p-values of the statistical tests to compare if both groups (controls vs. patients) matched in NART, age, level of education, or gender are presented in the last column. p-value at an alpha value of 0.05. Additional abbreviations to those defined in the main paper: LOC, Loss of Consciousness; PTA, Post-Traumatic Amnesia*.

### DTI Data Processing

**Figure 1:**
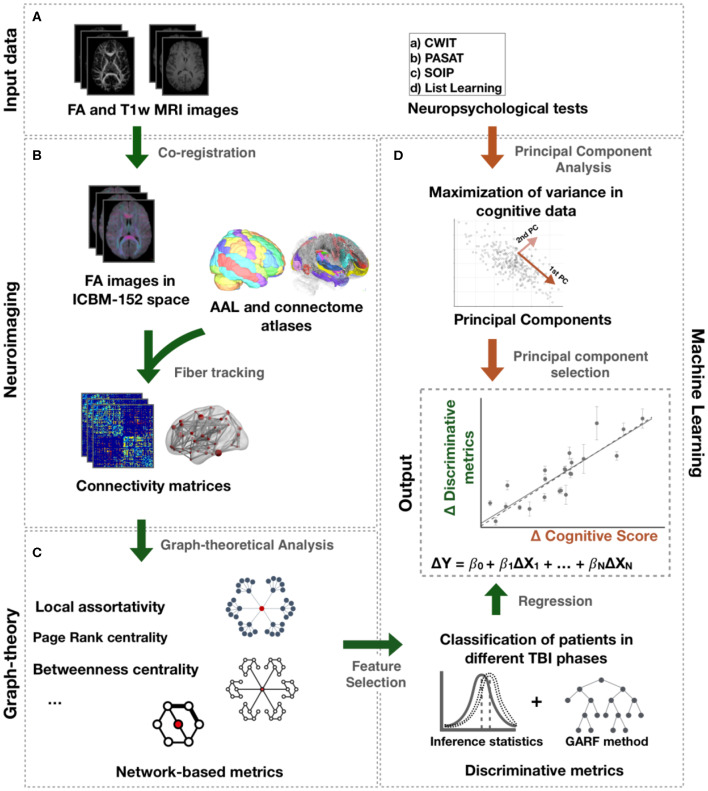
Processing pipeline. **(A)** Input data comprise FA and T1-weighted images and neuropsychological tests. **(B)** Connectome and AAL atlases were mapped to each individual FA image in ICBM-152 space. Mean FA along the tracts of the connectome atlas between the AAL regions was computed to create a connectivity matrix for each individual. **(C)** Network metrics were calculated from those matrices. **(D)** Statistical tests and genetic algorithm based random forest method (GARF) were used to classify patients in different TBI post-injury phases and, thus, identify the network metrics assessing changes following TBI. The change over time of those metrics, Δ*X*, were further investigated to explain changes in cognitive function, Δ*Y*, using regression models. The change in cognitive function, Δ*Y*, represents the change of the principal components obtained after applying PCA to the neuropsychological tests.

Fiber orientation reconstruction was carried out using FMRIB Diffusion Toolbox to create fractional anisotropic (FA) images. FA images were then normalized to standard space by a combination of linear and non-linear transformations (Figure 1) (16). All registrations were visually inspected. In standard space, tracts from the recently published structural connectome atlas space were projected to each individuals' FA image using DSI Studio (http://dsi-studio.labsolver.org) (17). This atlas contains 550,000 white-matter tracts verified manually by experienced neuroanatomists (17). Mean FA along the paths between each of the 90 regions of the AAL atlas were computed and saved in a connectivity matrix for each individual. This approach is inspired by a previous TBI study (18).

Network metrics were estimated from the connectivity matrices to quantify network integration, segregation, centrality, and resilience to perturbations (19). A detailed description of all network metrics is available in Table S4 in the Extended Data. Network integration and segregation measures describe the ability of a network to integrate information from distributed regions and the ability for local specialized information processing, respectively. Examples of those metrics are strength (the sum of all connection strengths to a given region), local clustering coefficient (measure the connectedness of neighbors of a region), local efficiency (the inverse of the average shortest path length of a node to their nodes), local assortativity (measure the contribution of a region to maintain the integration of information in the network after disruption), eigenvector and page rank centrality (measure the influence of a region has on a network), betweenness centrality (measure how often a region is traversed by the shortest paths in the network), and closeness centrality (measure the average shortest path of a region to all other regions in the network). More detailed descriptions are provided in references (19).

### Lesions Probability Map

Lesion masks were manually drawn for 19 patients by a neurosurgeon on the baseline scans, as described in Aribisala et al. (20). Lesion masks are defined as a binary mask where the lesioned voxels are labeled as 1 and the remaining voxels are labeled as 0. The masks include any visible contusion, hematoma, or edema. Visible lesions were not present for the remaining four patients. Lesion masks were then co-registered to the MNI-152 space using the warp fields of T1-MRI registration to standard space, which was conducted with linear and non-linear transformations in FSL (16).

To create a probability map, lesion masks were summed, and normalized to 0–100% range with 100% (0%) indicating the presence of a lesion in all (no) patients. Due to the heterogeneity of TBI, we applied a threshold to keep the most prevalent lesions and remove more sporadic ones. Therefore, only the lesions observed in at least three patients were considered.

We additionally calculated the Sørensen–Dice similarity coefficient to measure the overlap/similarity ratio of the Lesions Probability Map as an ROI with each AAL ROIs. This allowed us to inspect the impact of injuries to the observed changes in network metrics at a region level in our cohort.

### Components of Cognitive Function

Patients and controls underwent a full battery of standardized neuropsychological tests sensitive to cognitive impairments in mild TBI at each time of scanning. The tests included assessments of attention, memory, executive functions, and semantic knowledge. Due to missing data for some follow-up assessments, we restricted our analysis to the following tests: Speed of Information Processing (SOIP), Paced-Auditory-Serial-Addition Test (PASAT), D-KEFS Color-Word-Interference Test (CWIT), and List Learning (21–23). Early and late post-injury neuropsychological scores were compared with controls by inferential tests with false discovery rate (FDR) correction.

Following Irimia et al. (24) and Kuceyeski et al. (25), principal component analysis (PCA) was used to combine multiple cognitive scores in a set of principal components that maximize the variance of the neuropsychological tests and represent main cognitive functions. Since PCA is sensitive to skewness (26), cognitive scores with z-skewness higher than 1.96 were prior transformed using Box-Cox transformation (27).

### Network Metrics Selection and Association With Cognitive Function

We conducted a two-step feature selection to identify the most discriminative network metrics between different TBI phases using R (http://caret.r-forge.r-project.org/). This allowed us to identify a set of network metrics that changed over time and we interpreted as being causally related to the injury. Since the two-step feature selection was a cross-sectional analysis, a sample size of 46 was used (i.e., 23 samples for each TBI phase/time point or class).

Finally, using a regression model, we investigate how changes in those network metrics may be predictive of cognitive function over time.

#### Connectivity Metrics of MTBI

Initially, we used inferential tests to rank standardized network metrics that differed significantly between mTBI patients in the early and late post-injury phases. Following Thatcher et al. (28), Barik et al. (29), and Haury et al. (30), correction for multiple comparison was considered not relevant as the goal of this step was to reduce the number of features by separating the most significant metrics from the less significant and not draw inferential conclusion.

In the second step, a cross-sectional and multivariate study was conducted to select combinations of the previously selected metrics that best discriminate between patients in early or late post-injury time phases. To that end, a 5-fold cross-validation with genetic algorithm random forest (GARF) approach was used to classify patients in the different TBI phases. Although classification algorithms are often used to generate predictive models, our main intention was to identify the most discriminative network metrics between TBI phases. We opted to use GARF as it boosts the Random Forests performance while reducing considerably the number of metrics needed for classification (31).

#### Longitudinal Analysis

After identifying network metrics discriminating between different TBI phases, we investigated their association with cognitive score. This was achieved by a linear regression model, as described in the following equation:

(1) $$\begin{array}{l} \Delta Y=\beta_{0}+\beta_{1}\Delta X_{1}+\beta_{2}\Delta X_{2}+\cdots +\beta_{N}\Delta X_{N} \end{array}$$

where Δ represents the difference between late and early post-injury data for the *N* standardized network metrics (*X)* and the new neuropsychological score obtained by PCA (*Y)*. The fitting of the linear regression model was achieved using stats package in *R*, which uses the least-squares method to minimize the sum of the squares of residuals. Initially, we included all selected network metrics in the linear regression model. However, to overcome multicollinearity and remove unnecessary metrics, we discarded, at each round, the metric with the highest *p*-value, until the regression model and its coefficients were statistically significant.

### Experimental Design and Statistical Analysis

Inferential statistical tests were used to compare neuropsychological scores, PCA components, and network metrics for both groups. Depending on whether parametric assumptions were satisfied, paired *t*-test or Wilcoxon signed-rank and independent *t* or Mann–Whitney *U* were used at a significance level of 5%. In multiple comparisons, FDR correction was applied at a significance level of 5%.

The regression model was evaluated by normalized root mean square error (nRMSE), adjusted *R*^2^, and residuals inspection. To verify whether some of the underlying assumptions of regression had been violated, variance inflation factor (VIF) was used to identify multicollinearity among regression network metrics, and the inspection of residuals included the analysis of the scatter plot of residuals vs. predicted scores, lag, and normal probability plots of residuals. *F* test was used to test the null hypothesis that the fitting either using intercept-only model or our proposed regression model is the same. The rejection of the null hypothesis suggests a better fitting by the proposed model and its predictors.

The final regression model was investigated to examine the presence of bias, overfitting, and the significance of the network metrics selected by GARF. The former was achieved by randomly selecting 20 patients for training and three for testing. We ran this analysis 1,000 times and tested different split sizes (20–3, 18–5, 19–4, etc.). The significance of the network metrics selected by GARF was assessed by random selection of metrics followed by fitting of a linear regression model to predict changes in new neuropsychological score. The network metrics were selected from the set obtained in the first step of feature selection. We ran this analysis 1,000 times and, for each run, computed the nRMSE, and adjusted *R*^2^.

### Data Accessibility

Data and code will be made available after acceptance of the manuscript.

## Results

### Network-Based Metrics of MTBI

**Figure 2:**
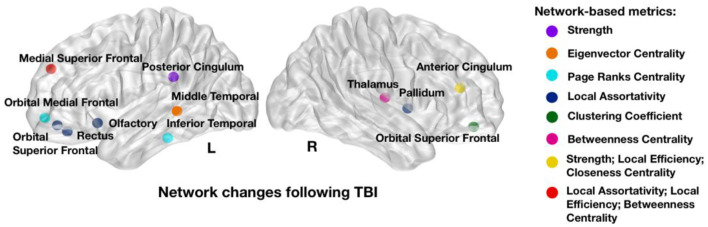
Network metrics that showed strongest power in discriminating between early and late post-injury time phases: strength in left posterior and right cingulum, Eigenvector centrality in left middle temporal; Page Ranks centrality in left inferior temporal and left orbital medial frontal; Local Assortativity in right pallidum, left rectus, left orbital superior frontal, left olfactory, and left medial superior frontal; Clustering Coefficient in right orbital superior frontal; Betweenness Centrality in right thalamus and left medial superior frontal; Local Efficiency in right anterior cingulum and left medial superior frontal and Closeness Centrality in left medial superior frontal. Colors illustrate the network metrics identified in each region. L, Left Side; R, Right Side.

The GARF model selected a total of 16 network metrics involving 12 brain regions and showed a 5-fold cross-validation accuracy of 83.3%, confirming robust discrimination between early and late post-injury time phases. Figure 2 shows the brain regions and properties that were selected by the model, with a majority located in the fronto-temporal cortex.

### Cognitive Function Assessment

**Figure 3:**
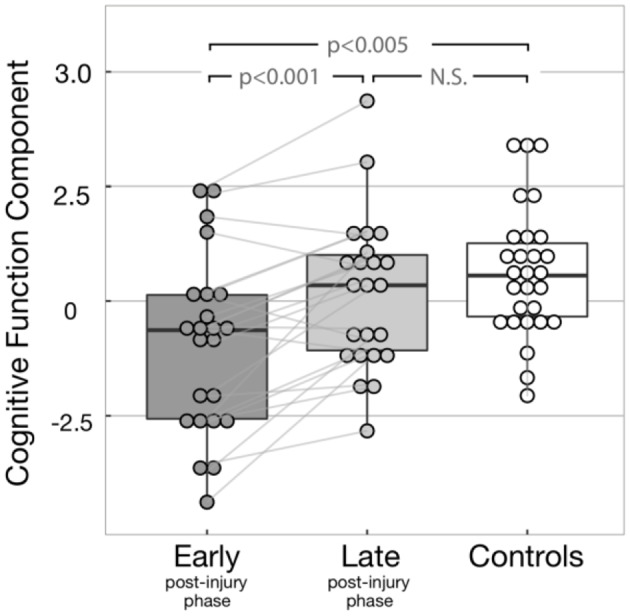
Cognitive function component (CFC) for all subjects. The light gray lines display the change of CFC between early and late post-injury phases display for each patient. Significant differences were found between early post-injury CFC and both controls (*p*-value = 0.001) and late post-injury CFC (*p*-value < 0.001). As expected, no significant difference was observed between controls and late post-injury CFC (*p*-value = 0.268), due to the recovery of most patients.

Inferential statistics were used to compare cognitive functioning between patients in early or late post-injury phase along with controls (Figure 3). Cognitive functioning was assessed by PCA to reduce Type 1 errors commonly found in multiple statistical comparisons. This approach resulted in one main component, explaining 62.3% of the variance. PASAT and CWIT showed the highest weightings/contributions to the first PCA component, supporting the interpretation that the first PCA component predominantly represents executive functions. For ease of interpretation, we will refer to the first PCA component as cognitive function component (CFC). In our recent study, CFC showed to be correlated with a multivariate measure for intrinsic injury severity (32). An increase over time in CFC suggests an overall improvement of cognitive function. A total of 18 patients showed an increase in cognitive functioning at 12 months, four showed a low decay, while one did not show change in its cognitive functioning over 1 year.

Patients did not show significant differences in the CWIT inhibition trial when compared with controls. In contrast, they showed significant disability in reading and naming colored patches, as quantified by the baseline trials of CWIT. Since reading and naming also involve cognitive pathways (33, 34), we include those measures in the PCA.

### Longitudinal Relationships Between Network-Based Metrics of MTBI and Cognitive Functions

**Figure 4:**
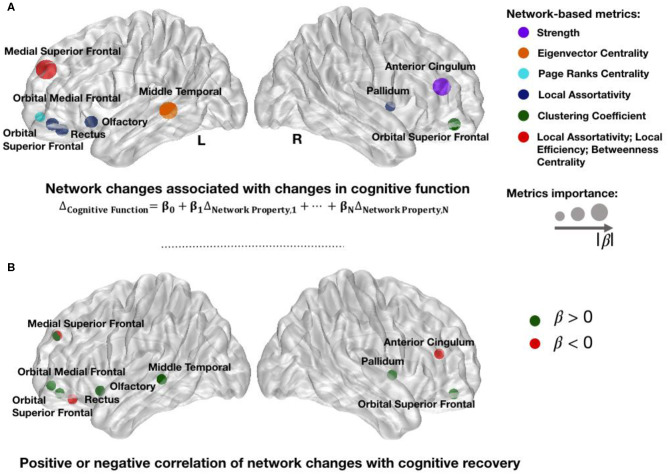
Changed network metrics associated with cognitive improvement. Colors in **(A)** illustrate the network metrics identified in each region and the circles' radius indicates the importance for prediction based on the standardized coefficients of regression model. The list of network changes associated with changes in cognitive function were strength in right cingulum; eigenvector centrality in left middle temporal; page ranks centrality in left orbital medial frontal; local assortativity in right pallidum, left rectus, left orbital superior frontal, left olfactory, and left medial superior frontal; clustering coefficient in right orbital superior frontal; betweenness centrality in left medial superior frontal; and local efficiency in left medial superior frontal cortices. Red circles in **(B)** represent regions in which an increase in their network metrics is negatively correlated with recovery. Possible justifications to these changes are secondary brain injuries, inability to recover after the trauma, or brain maladaptation. Green circles in **(B)** illustrate the regions in which an increase in their network metrics over 1 year is positively correlated with improvement, therefore suggesting brain adaptation or recovery. L, Left Side; R, Right Side.

After identifying the network metrics associated with mTBI, we performed a longitudinal analysis to investigate associated changes in cognitive performance, namely, in CFC. A significant multiple linear regression equation (*F* test: *p* < 0.0001) was achieved by a total of 11 significant network metrics between nine brain regions. All metrics had an acceptable (35) VIF lower than 10. Figure 4A shows the 11 network metrics with the greatest power in explaining changes in cognitive function and their correlation sign with recovery (Figure 4B). The importance of these 11 features to explain changes in cognitive function is ranked based on the standardized coefficients of the regression model, which are available in Table S5 in Extended Data. From the 11 network metrics identified by the model, 45% measured local assortativity, and the most important network change to explain changes in cognitive function was local assortativity in left medial superior frontal. For most of the network metrics, late post-injury patients in our study showed higher or lower median values than controls. However, an increase on the network metrics was observed early after the injury in anterior cingulate cortex (ACC) and gyrus rectus (GR), followed by a normalization to controls values in the late post-injury phase. The most common of the network metrics assesses local assortativity. Global assortativity quantifies the tendency of regions to be connected to regions with similar strength. A network with positive assortativity is likely to maintain its ability to integrate information after disruption (i.e., it is more resilient to insults), as it may contain interconnected high-strength regions (19). Local assortativity measures the contribution of each node to network global assortativity (36).

**Figure 5:**
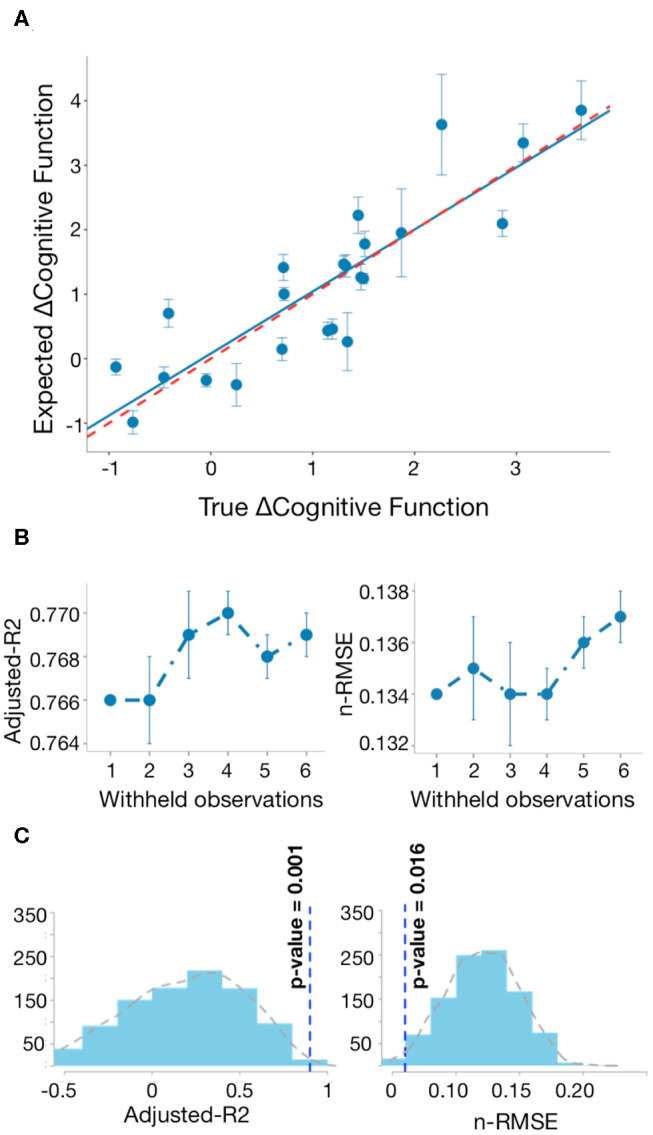
Regression model of changed network metrics explains changes in cognitive function. **(A)** Bootstrap line (blue) closely approximates to the ideal line in red (adjusted *R*^2^ = 76%), when observed and expected responses are equal. The vertical lines indicate the prediction standard deviation for each patient across 1,000 runs. **(B)** Bootstrap analysis: for each different split size, we saved the mean predictions after 1,000 runs and repeated this process 10 times. The vertical lines indicate the standard deviation of the adjusted *R*^2^ and nRMSE after repeating the bootstrap analysis 10 times. **(C)** Distribution of adjusted *R*^2^ and nRMSE by taking random selection of different sets of discriminative network metrics between early and late post-injury stages 1,000 times.

The linear regression equation proposed in this study showed an adjusted *R*^2^ of 90% and nRMSE of 0.06. These results indicate that the model explains 90% of the variability found in the CFC over time in patients. Figure 5A shows the bootstrap line close to the ideal line, when the observed and expected responses are equal. This result implies consistency of our model performance and no presence of overfitting. Similar results were achieved with different split sizes (Figure 5B). Figure 5C shows the distribution of nRMSE and adjusted *R*^2^ by taking random selections of different sets of 11 network metrics after the first feature selection step. The vertical lines correspond to the results of the significant regression equation. The low *p*-values suggest that network metrics in Figure 4A are associated with changes following TBI and not random variability.

**Figure 6:**
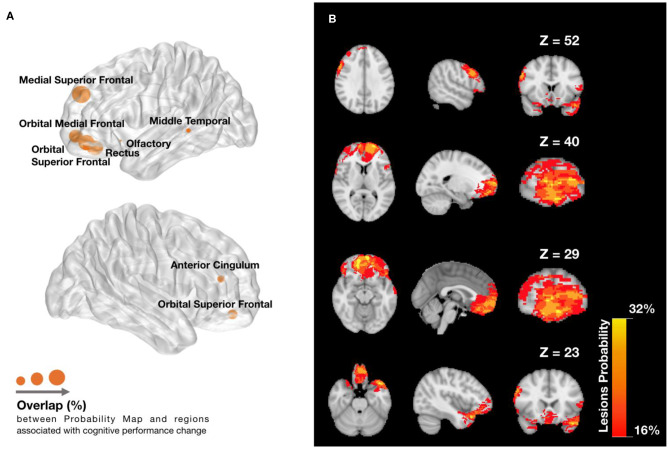
Lesions overlap with **(A)** regions associated with cognitive function component (CFC) change and **(B)** across patients. **(A)** Dice overlap between Lesions Probability Map in **(B)** and AAL regions found to be predictive of changes in cognitive function. Node radius indicates the amount of Dice overlap for the predictive AAL regions, which can be found in extended data (Figure 4). **(B)** Lesions Probability Map for different *Z* voxel coordinates of MNI space, showing the visible overlapping amount across patients. The lesions are found mainly in the frontal lobe, temporal lobe, and slightly in the anterior cingulate cortex. The colormap ranges between an overlap of 16 and 32% patients.

Figure 6A shows the Dice overlap of the AAL regions assessed by the 11 network metrics with the Probability Map of Lesions (Table S5; Extended Data). Although most of the areas showed overlap, frontal brain areas were particularly affected (Figure 6B).

## Discussion

In this preliminary study of 23 patients, we investigated the existence of longitudinal relationships between changes in structural connectivity and changes in cognitive function following TBI. Using graph theory and a regression model, we identified 11 changes in structural connectivity explaining 90% of the observed variability in cognitive function change over 1 year. The observed brain reorganization suggests possible involvement of frontal regions and the anterior cingulate cortex in cognitive function recovery. The regression model also combines information from other brain regions, emphasizing the value of graph-theoretical network-wide perspectives in cognitive neuroscience.

Limitations of our work include the relatively small size of our dataset, which reflects the challenges of recruiting large longitudinal cohorts with repeated clinical and imaging data. Furthermore, the patient imaging and psychological data were acquired at only two time points, within days of injury and a year later, so we could not establish the starting and stopping point for each different reorganization pattern or its causality. Another limitation in this study is the resolution of DTI images, which could negatively affect fiber tracking. We overcome it by a two-step co-registration followed by quantification of FA values along a connectome atlas. The pathways comprised in this atlas were manually examined and labeled by experienced neuroanatomists (17). A similar approach has also been used in previous severe TBI studies (18). Finally, our dataset comprises patients with both mild and moderate TBI, which could potentially prejudice our understanding about recovery in mild TBI. Using the same dataset, Croall et al. (15) did not observe any significant differences between patients with mild or moderate TBI in any diffusion metrics. Since only one moderate patient had a GCS lower than 10 and the majority had 12, the moderate group is close to the milder end of injuries. As in Croall et al. (15), we assume that these patients have microstructural changes similar to mild injuries.

### Areas Associated With Cognitive Function

The regions identified by our regression model are consistent with previous cognitive function localization literature (37–42). For instance, Zhou et al. (43) found WM volume loss in the left and right rostral anterior cingulum to be correlated with changes in memory and attention. Furthermore, left medial superior frontal cortex (MSFC), gyrus rectus (GR), and middle temporal cortex (MTC) are known to be associated with specific tasks involved in the neuropsychological tests assessed in this study, such as auditory verbal attention, scores on sequencing, semantic retrieval, semantic memory, and semantic control (44–47). Changes in the left olfactory cortex (OC) also contributed to the prediction model: to our knowledge, no cognitive functions are directly supported by it. Although two patients lost sense of smell, the OC has connections to both orbitofrontal cortex and basal ganglia and is implicated in executive function pathways.

### Network Topology Changes Over Time After MTBI

**Figure 7:**
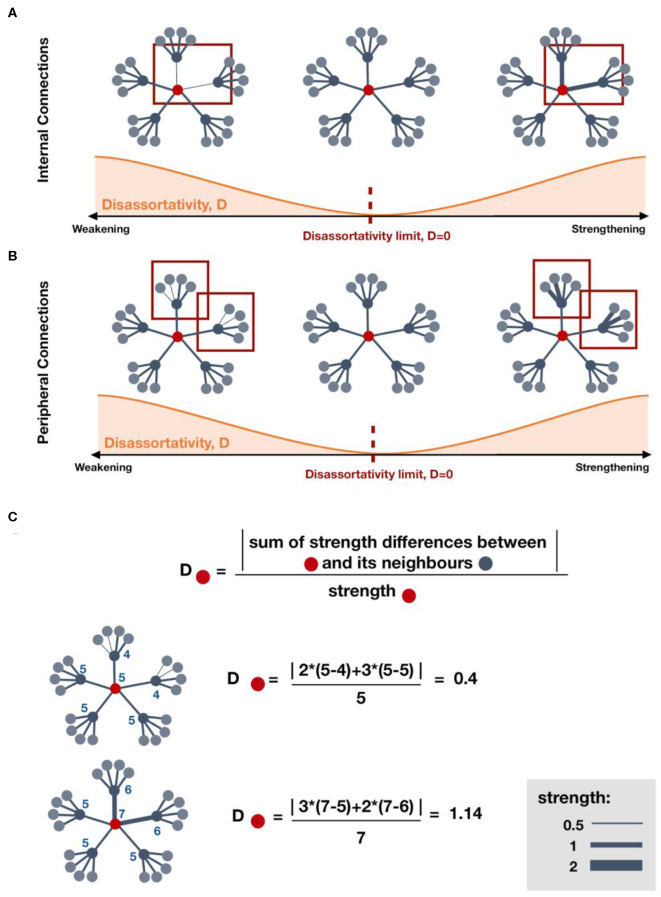
Effect on local disassortativity by changes in regions' strength. **(A,B)** indicate some possible mechanisms of local disassortativity change. **(A)** Local disassortativity of the red region decreases when the strength differences between red regions and its neighboring regions is reduced. This reduction may occur by strengthening or weakening of internal connections. When disassortativity decreases, the red region is more assortative. **(B)** Similarly, local disassortativity decreases with peripheral weakening or peripheral strengthening. When the red region is connected to other regions with lower strength, disassortativity may decrease by strengthening of its low-strength neighbors. However, when connected to regions with higher strength, strengthening of those neighbors will increase disassortativity. **(C)** Examples on how to calculate local disassortativity for regions colored in red.

The network metric changes observed in MTC, pallidum, and OC showed a positive correlation with ΔCFC (Figure 4B). Those changes suggest strengthening to regions that are highly connected, which may be a brain response to improve or establish a “rich-club organization” for information integration (9), either due to local lesions or disruptions in cognitive subnetworks. Since the pallidum is highly connected to other high-connected regions in the brain (39), the increase in local assortativity suggests that it connects to other similar regions, preferably high-strength regions (Figure 7A). Similarly, a positive correlation between an increase of local assortativity in OC and ΔCFC may indicate a strengthening of its neighbors' connections involved in executive function circuits, such as orbitofrontal and basal ganglia (Figures 7B,C) (40). Some possible explanations for network metric changes, which are negatively correlated with ΔCFC, may be continuous deterioration, incapacity to recover, maladaptive plasticity, or compensatory pathways recruitment, which are no longer required after brain recovery. Increased local assortativity in MSFC was observed to be negatively correlated with ΔCFC (Figure 4B). Since the MSFC is highly susceptible to injury (41), internal disconnections may contribute to the reduction in the average strength differences, which leads to increased local assortativity (Figures 7A,C).

We observed an increase in early post-injury phase in anterior cingulate cortex strength and gyrus rectus local assortativity, followed by a decrease in 1 year. This is in agreement with a longitudinal study by five, who also observed increased structural connectivity in a subnetwork within 7 days post-injury in mTBI patients, including pathways from or to anterior cingulate cortex, and gyrus rectus.

### Conclusion

Our findings demonstrate a longitudinal relationship between changes in structural brain connectivity and changes in cognitive functions following TBI. The detailed graph theoretical analysis suggests that a combination of different network metrics in distinct brain regions captures most of the longitudinal variance in cognitive performance.

Future longitudinal investigations should assess patients' cognitive outcome and brain networks at more time points to enable personalized predictions of optimal rehabilitation strategies based on network metrics changes at higher temporal resolution.

## Data Availability Statement

Data and code to reproduce main figures can be found at: https://zenodo.org/record/3824190.

## Ethics Statement

The studies involving human participants were reviewed and approved by Newcastle University Ethics Committee. The patients/participants provided their written informed consent to participate in this study.

## Author Contributions

NM: study design and concept, analysis and interpretation of data, statistical analysis, and drafting the manuscript. CC: data acquisition and lesions masks drawing. AB: data acquisition and revised the manuscript. RF: study design and concept, interpreted the data, and revised the manuscript. PT: study design and concept, interpreted the data, imaging processing, and revised the manuscript.

## Conflict of Interest

The authors declare that the research was conducted in the absence of any commercial or financial relationships that could be construed as a potential conflict of interest.
