# Supplementary material for: Investigating Brain Network Changes and Their Association With Cognitive Recovery After Traumatic Brain Injury: A Longitudinal Analysis
Source: Front Neurol. 2020 Jun 9;11:369. doi: 10.3389/fneur.2020.00369 (PMC7296134; doi:10.3389/fneur.2020.00369)
Supplement: Supplementary file 1 [file Data_Sheet_1.docx]

**Supplementary material: Investigating brain network changes and their association with cognitive recovery after Traumatic Brain Injury: a longitudinal analysis**

**Network reorganization in traumatic brain injury**

**Nádia Moreira da Silva, MSc^1^, Christopher J. A. Cowie, MD^2,4^, Andrew M. Blamire, PhD^3^, Rob Forsyth, PhD^3^, Peter Neal Taylor, PhD ^1,5*^**

**Table S1**. Results of neuropsychological tests and first component of PCA of controls and patients in the early or late post-injury phase. The results of inferential statistical tests, to compare groups cognitive performance, are presented in the last columns.

| Test | Controls | Early post-injury patients | Late post-injury patients | Late post-injury patients vs. Controls | Early post-injury patients vs. Controls | Early post-injury vs. Late post-injury patients |
| --- | --- | --- | --- | --- | --- | --- |
| SOIP (motor speed adjusted) | Med = 75.9  IQR = 61.1-92.1 | Med = 57.7  IQR = 45.6-70.8 | Med = 70.2  IQR = 56.9-79.4 | *p* = 0.294  *p_FDR_* = 0.665 | ***p* = 0.009**  ***p_FDR_* = 0.030** | ***p* = 0.004**  ***p_FDR_* = 0.010** |
| PASAT (2 second intervals, percentage correct) | Med = 57  IQR = 44.3-68.0 | Med = 45  IQR = 4.0-58.5 | Med = 48  IQR = 45.0-58.5 | *p* = 0.532  *p_FDR_* = 0.665 | ***p* = 0.006**  ***p_FDR_* = 0.030** | *p* = 0.059  *p_FDR_* = 0.118 |
| List Learning (final trial score) | Med = 12  IQR = 10-13 | Med = 10  IQR = 5.5-12.0 | Med = 11  IQR = 9.5-13.0 | *p* = 0.498  *p_FDR_* = 0.665 | ***p* = 0.004**  ***p_FDR_* = 0.030** | ***p* = 0.002**  ***p_FDR_* = 0.010** |
| Digit Span Backwards (sequence length) | Med = 7  IQR = 6-8 | Med = 6  IQR = 5.0-8.5 | Med = 6  IQR = 5-8 | *p* = 0.116  *p_FDR_* = 0.627 | *p* = 0.101  *p_FDR_* = 0.168 | *p* = 0.569  *p_FDR_* = 0.632 |
| Spatial Span Backwards (sequence length) | Med = 8  IQR = 7-9 | Med = 7  IQR = 6.0-8.5 | Med = 7  IQR = 6-8.5 | *p* = 0.379  *p_FDR_* = 0.665 | *p* = 0.161  *p_FDR_* = 0.179 | *p* = 0.397  *p_FDR_* = 0.567 |
| Verbal Letter Fluency (Total correct) | Med = 40.5  IQR = 34.0-47.3 | Med = 36  IQR = 27.0-46.5 | Med = 40  IQR = 32-45 | *p* = 0.927  *p_FDR_* = 0.927 | *p* = 0.146  *p_FDR_* = 0.179 | *p* = 0.086  *p_FDR_* = 0.143 |
| Category Fluency (Total correct) | Med = 43.5  IQR = 39.8-45.3 | Med = 41  IQR = 35.5-45.0 | Med = 41  IQR = 33.5-44.5 | *p* = 0.188  *p_FDR_* = 0.627 | *p* = 0.129  *p_FDR_* = 0.179 | *p* = 0.512  *p_FDR_* = 0.632 |
| C-WIT (colour naming, completion time) | Med = 27  IQR = 25.8-30.5 | Med = 32  IQR = 27.5-36.5 | Med = 29  IQR = 26.5-37.5 | *p* = 0.175  *p_FDR_* = 0.627 | ***p* = 0.019**  ***p_FDR_* = 0.048** | ***p* = 0.015**  ***p_FDR_* = 0.038** |
| C-WIT (word naming, completion time) | Med = 21.5  IQR = 19.0-25.0 | Med = 24  IQR = 22-28 | Med = 22  IQR = 20-25 | *p* = 0.497  *p_FDR_* = 0.665 | ***p* = 0.024**  ***p_FDR_* = 0.048** | ***p* = 0.010**  ***p_FDR_* = 0.033** |
| C-WIT (inhibition, completion time) | Med = 55  IQR = 48.8-58.5 | Med = 54  IQR = 43.5-60.0 | Med = 51  IQR = 44-66 | *p* = 0.684  *p_FDR_* = 0.760 | *p* = 0.865  *p_FDR_* = 0.865 | *p* = 0.976  *p_FDR_* = 0.976 |
| 1^st^ Component PCA | Med = 0.55  IQR = -0.34-1.26 | Med = -0.64  IQR = -2.57-0.13 | Med = 0.34  IQR = -1.08-1.00 | *p* = 0.268  *p_FDR_* = 0.268 | ***p* = 0.001**  ***p_FDR_* = 0.001** | ***p* <0.001**  ***p_FDR_* <0.001** |

^For these columns, a bold^ *^p^* ^value indicates it is significant at an alpha value of 0.05. P-values following FDR correction are displayed as p^_FDR_^.^

**Table S2**. Clinical data of each patient assessed in this study.

| Patient | Age | Sex | Level of Education | NART | TBI | Injury cause | GCS | LOC | PTA |
| --- | --- | --- | --- | --- | --- | --- | --- | --- | --- |
| 001 | 24 | M | 9 | 81.00 | moderate | Unclear | 10 | 1.00 | 1 |
| 002 | 53 | M | 9 | 105.00 | moderate | Assault | 12 | 15.00 | 4 |
| 003 | 23 | F | 9 | 105.00 | moderate | Fall | 12 | 0.00 | 1 |
| 004 | 38 | M | 14 | 105.00 | moderate | Fall | 12 | 3.00 | 336 |
| 005 | 65 | F | 14 | 122.00 | moderate | Fall | 9 | 10.00 | 240 |
| 006 | 65 | M | 14 | 127.00 | mild | Fall | 14 | 5.00 | 2 |
| 007 | 18 | M | 9 | 126.00 | mild | Accident | 14 | 5.00 | 0 |
| 008 | 22 | M | 9 | 84.00 | mild | RTA^1^ | 15 | 0.00 | 0 |
| 009 | 64 | M | 9 | 90.00 | mild | Fall | 14 | 10.00 | 0 |
| 010 | 40 | F | 14 | 118.00 | mild | Fall | 14 | 0.00 | 2 |
| 011 | 47 | M | 9 | 106.00 | mild | Assault | 15 | 3.00 | 0 |
| 012 | 36 | M | 9 | 102.00 | mild | Assault | 14 | 5.00 | 0 |
| 013 | 24 | M | 11 | 105.00 | mild | Assault | 15 | 1.00 | 0 |
| 014 | 35 | F | 15 | 117.00 | mild | Fall | 13 | 5.00 | 30 |
| 015 | 33 | F | 9 | 90.00 | mild | Fall | 15 | 1.00 | 1 |
| 016 | 22 | M | 9 | 84.00 | mild | RTA^1^ | 14 | 0.00 | 1 |
| 017 | 66 | M | 9 | 106.00 | mild | Fall | 15 | 0.00 | 0 |
| 018 | 46 | M | 11 | 111.00 | mild | Accident | 15 | 1.00 | 0 |
| 019 | 34 | M | 9 | 92.00 | mild | Assault | 15 | 1.00 | 0 |
| 020 | 26 | M | 14 | 94.00 | mild | Assault | 14 | 0.00 | 7 |
| 021 | 49 | M | 9 | 97.00 | mild | Fall | 15 | 0.00 | 0 |
| 022 | 58 | M | 14 | 117.00 | mild | Fall | 14 | 1.00 | 1 |
| 023 | 29 | M | 17 | 113.00 | mild | Sport | 14 | 0.50 | 6 |

^1^ RTA – Road Traffic Accident

**Table S3.** Clinical data of each control assessed in this study.

| Control | Age | Sex | Level of Education | NART |
| --- | --- | --- | --- | --- |
| 001 | 19 | M | 9 | 86.00 |
| 002 | 66 | M | 9 | 105.00 |
| 003 | 28 | F | 15 | 113.00 |
| 004 | 30 | M | 14 | 123.00 |
| 005 | 35 | M | 9 | 115.00 |
| 006 | 63 | M | 9 | 107.00 |
| 007 | 25 | M | 16 | 107.00 |
| 008 | 59 | M | 11 | 115.00 |
| 009 | 54 | F | 9 | 121.00 |
| 010 | 59 | M | 9 | 106.00 |
| 011 | 22 | F | 11 | 97.00 |
| 012 | 48 | M | 9 | 117.00 |
| 013 | 45 | M | 11 | 107.00 |
| 014 | 21 | F | 9 | 107.00 |
| 015 | 19 | M | 11 | 112.00 |
| 016 | 65 | M | 9 | 115.00 |
| 017 | 36 | M | 14 | 115.00 |
| 018 | 35 | M | 14 | 117.00 |
| 019 | 28 | M | 14 | 113.00 |
| 020 | 54 | M | 9 | 115.00 |
| 021 | 37 | M | 9 | 112.00 |
| 022 | 50 | M | 9 | 100.00 |
| 023 | 38 | F | 9 | 96.00 |
| 024 | 19 | M | 14 | 121.00 |
| 025 | 48 | M | 14 | 117.00 |
| 026 | 20 | M | 14 | 106.00 |
| 027 | 41 | M | 9 | 113.00 |
| 028 | 42 | F | 11 | 111.00 |

**Table S4.** List presenting the network metrics used in this study to quantify network segregation, integration, resilience and centrality.

| **Segregation** | Transitivity |
| --- | --- |
|  | Average Clustering Coefficient |
|  | Local Clustering Coefficient |
|  | Local Efficiency |
|  | Small-worldness |
| **Integration** | Characteristic Path Length |
|  | Global Efficiency |
|  | Small-worldness |
| **Resilience** | Assortativity Coefficient |
|  | Local Assortativity |
| **Centrality** | Closeness |
|  | Betweenness |
|  | Eigenvector |
|  | Page-Ranks |
|  | Eccentricity |
| **Basic** | Strength |
|  | Diameter |

**Table S5.** Standardized coefficients and p-values of eleven network metrics between 9 different regions, which change was predictive of cognitive performance following mTBI. The last column present the Dice overlap of the Lesions Probability Map with the eleven network metrics.

| AAL Region | Network metric | Coefficient | p-value | Dice Overlap |
| --- | --- | --- | --- | --- |
| Right Anterior Cingulum | Strength | -1.89 | <0.001 | 0.06 |
| Right Orbital Superior Frontal | Clustering Coefficient | 0.74 | 0.003 | 0.11 |
| Left Medial Superior Frontal | Local Efficiency | 1.33 | <0.001 | 0.15 |
| Left Orbital Superior Frontal | Local Assortativity | 0.96 | <0.001 | 0.14 |
| Left Olfactory | Local Assortativity | 0.84 | <0.001 | 0.01 |
| Left Medial Superior Frontal | Local Assortativity | -2.02 | <0.001 | 0.15 |
| Left Rectus | Local Assortativity | -0.85 | <0.001 | 0.14 |
| Right Pallidum | Local Assortativity | 0.47 | 0.005 | 0 |
| Left Medial Superior Frontal | Betweenness centrality | 0.46 | 0.02 | 0.15 |
| Left Middle Temporal | Eigenvector centrality | 1.96 | <0.001 | 0.02 |
| Left Orbital Medial Frontal | PageRanks centrality | 0.36 | 0.004 | 0.14 |
